# CuO-NPs Induce Apoptosis and Functional Impairment in BV2 Cells Through the CSF-1R/PLCγ2/ERK/Nrf2 Pathway

**DOI:** 10.3390/toxics13040231

**Published:** 2025-03-21

**Authors:** Linhui Yang, Lina Zhu, Bencheng Lin, Yue Shi, Wenqing Lai, Kang Li, Lei Tian, Zhuge Xi, Huanliang Liu

**Affiliations:** 1 College of Oceanography and Ecological Science, Shanghai Ocean University, Shanghai 201306, China; ylh321@outlook.com (L.Y.); zlnjuliana@163.com (L.Z.); 2 Military Medical Sciences Academy, Academy of Military Sciences, Tianjin 300050, China; linbencheng123@126.com (B.L.); october2144@163.com (Y.S.); laiwenqing@126.com (W.L.); tjlikang@126.com (K.L.); tjtianlei@126.com (L.T.)

**Keywords:** CuO-NPs, microglia, cell apoptosis, CSF-1R/PLCγ2/ERK/Nrf2 pathway, functional impairment

## Abstract

Copper oxide nanoparticles (CuO-NPs) induce neurological diseases, including neurobehavioral defects and neurodegenerative diseases. Direct evidence indicates that CuO-NPs induce inflammation in the central nervous system and cause severe neurotoxicity. However, the mechanism of CuO-NP-induced damage to the nervous system has rarely been studied, and the toxicity of different CuO-NP particle sizes and their copper ion (Cu^2+^) precipitation in microglia (BV2 cells) is worth exploring. Therefore, this study investigated CuO-NPs with different particle sizes (small particle size: S-CuO-NPs; large particle size: L-CuO-NPs), Cu^2+^ with equal molar mass (replaced by CuCl_2_ [Equ group]), and Cu^2+^ precipitated in a cell culture solution with CuO-NPs (replaced by CuCl_2_ [Pre group]), and examined the mechanism of action of each on BV2 microglia after co-culture for 12 h and 24 h. The activity of BV2 cells decreased, the morphology was damaged, and the apoptosis rate increased in all the exposed groups. Toxicity increased time- and dose-dependently, and was highest in the Equ group, followed by the S-CuO-NPs, L-CuO-NPs, and Pre groups, respectively. Subsequently, we investigated the mechanism of S-CuO-NP-induced cell injury, and revealed that S-CuO-NPs induced oxidative stress and inflammatory response and increased the membrane permeability of BV2 cells. Moreover, S-CuO-NPs reduced the ratio of p-CSF-1R/CSF-1R, p-PLCγ2/PLCγ2, p-extracellular signal-regulated kinase (ERK)/ERK, p-Nrf2/Nrf2, and Bcl-2/Bax protein expression in microglia, and elevated cleaved caspase-3 expression. The CSF-1R/PLCγ2/ERK/Nrf2 apoptotic pathway was activated. The downregulation of CX3CR1, CSF-1R, brain-derived neurotrophic factor (BDNF), and IGF-1 protein expression indicates impairment of the repair and protection functions of microglia in the nervous system. In summary, our results reveal that CuO-NPs promote an increase in inflammatory molecules in BV2 microglia through oxidative stress, activate the CSF-1R/PLCγ2/ERK/Nrf2 pathway, cause apoptosis, and ultimately result in neurofunctional damage to microglia.

## 1. Introduction

Copper oxide nanoparticles (CuO-NPs) are popular toxic substances. CuO-NPs have various physical and chemical properties from large-size copper oxide, and are different from ordinary copper oxide particles because their particle size is at the junction of macroscopic and microscopic objects. Their light, electricity, sound, force, heat, magnetic activity, internal pressure, chemical activity, and catalytic activity exhibit great changes, due to their excellent physical and chemical properties. They are widely used in many industries, such as photoelectric products, the chemical industry, and biomedicine. CuO-NPs exhibit a smaller particle size than ordinary copper oxide, and the particle size is directly related to many chemical properties, including specific surface area, solubility, and reactivity [1], which strongly affect the toxicity of nanoparticles. The small size characteristics of the nanoparticles help them to easily enter the organism, penetrate the biological barrier, and accumulate in the body. Further, the resulting large specific surface area increases the reactivity and interaction between the nanoparticles and biomolecules [2]. Therefore, CuO-NPs demonstrate high chemical activity and intrinsic toxicity.

Neurodegenerative diseases are characterized by a progressive loss of neuronal and central nervous system (CNS) function, and most neurodegenerative diseases currently have no cure [3]. Microglia, as immune sentinels of the CNS, are responsible for eliminating microorganisms, dead cells, excess synapses, protein aggregates, and other particles and soluble antigens that may harm the CNS [4,5,6,7]. This immunosurveillance function is critical for maintaining neuronal health and preventing neurodegenerative diseases [8]. However, sustained microglia overactivation induces a chronic neuroinflammatory response [9], and microglia-mediated neuroinflammation is a predominant feature of many neurodegenerative diseases, including Alzheimer’s disease (AD) [10], Parkinson’s disease (PD) [11], amyotrophic lateral sclerosis [12], etc. After activation, microglia release a large number of inflammatory mediators, such as TNF-α and IL-1β [13], which activate the death receptor pathway in neuronal cells, including caspase family members, thereby triggering a cascade reaction, causing the programmed death of neurons [14]. Normal immune surveillance and inflammatory regulation functions are destroyed as the microglia are damaged, and an excessive amount of reactive oxygen species (ROS) is produced, causing oxidative stress [15]. This induces cellular damage, impairs synaptic pruning and regeneration, and compromises neural circuit reconstruction, resulting in obstacles in neural circuit reconstruction, affecting learning, memory, and cognitive functions [16], and ultimately increasing the risk of neurodegenerative diseases such as AD and PD [17,18,19].

Although direct environmental monitoring data of CuO-NPs are scarce, CuO-NPs are highly toxic compared to other metal oxide nanoparticles, as well as carbon nanoparticles and carbon nanotubes [20]. CuO-NP exposure damages the liver, kidneys, lungs, and other organs [21,22,23]. CuO-NPs precipitate Cu^2+^ in a biological medium. Studies have demonstrated that dissolved Cu^2+^ derived from CuO-NPs can adsorb onto bacterial cell walls and translocate across the cell membrane into the cytoplasm, potentially disrupting intracellular processes [24]. Cu^2+^ reacts with the amino, sulfhydryl, and carboxyl groups to change the helix structure of bacterial proteins and inactivate the proteins, thereby killing bacteria. Concurrently, Cu^2+^ accumulates valence transitions in the catalytic Fenton or Haber–Weiss reaction, produces numerous hydroxyl radicals, induces oxidative stress, damages cell components, such as membrane lipids, proteins, and nucleic acids, and produces biological toxicity [25,26,27]. CuO-NPs, in addition to their ability to release Cu^2+^, exert a more pronounced biotoxic effect than the toxicity caused by the CuO-NP-precipitated Cu^2+^ [28]. High concentrations of reactive oxygen species caused by nanoparticles, such as peroxidizing free radicals, hydroxyl free radicals, and hydrogen peroxide, cause oxidative damage and destroy cell metabolism [29]. Further, they exert other effects associated with oxidative stress, such as membrane damage, cell necrosis, and autophagy [30,31,32]. The brain, as the most crucial organ of the human body, contains the most difficult blood–brain barrier (BBB) to penetrate, but nanoparticles can pass through the BBB and damage the brain [33].

At present, research on the toxic effects of CuO-NPs is mostly focused on their damage to animals and plants. Further, in vitro experiments are focused on their damage to non-nerve cells [34,35,36,37,38], and studies on their toxic effects and mechanisms on nerve cells, especially microglia, are very rare. The toxicity of CuO-NPs with different particle sizes and Cu^2+^ on microglia remains unknown. Therefore, this study investigated the damage and mechanism of CuO-NPs with different particle sizes, Cu^2+^ with equal molar mass (replaced by CuCl_2_), and CuO-NP-precipitated Cu^2+^ in cell culture media. The role of the CSF-1R/PLCγ2/ERK/Nrf2 signaling pathway in CuO-NP-induced apoptosis and neurologic damage of microglia were discussed to further analyze the toxic effect of CuO-NPs on microglia and their mechanism.

## 2. Materials and Methods

### 2.1. Preparation and Characterization of CuO-NP Suspension

Sigma-Aldrich supplied the CuO-NPs used in this study. CuO-NPs were suspended in normal saline separately, mixed, and vortex-mixed. The solution was then ultrasonically treated at 60 Hz and 4 °C for 4 h, and vortex-mixed every 10 min to prepare the CuO-NP suspension, which was stored at 4 °C. The particle size of the CuO-NPs was observed with a TEM instrument (Thermo Fisher, Waltham, MA, USA).

### 2.2. Cu^2+^ Precipitation

The CuO-NP-contaminated cell suspensions of small particle size, with different concentrations, were oscillated in an oscillating incubator (120 rpm) at 37 °C for 24 h, and centrifuged (3000 rpm) for 30 min. A 0.45 μm filter membrane was then used to filter the supernatant three times. The Cu^2+^ level in the supernatant was identified using inductively coupled plasma mass spectrometry (ICP-MS) (Model: AA-IE-280), which was repeated three times per sample.

### 2.3. Preparation of Copper Chloride

CuCl_2_·2H_2_O_2_ solid was dissolved in deionized water to prepare a 1 M stock solution (206.5 g/L). The dissolution was assisted by sonication, and then the volume was made up to 1 L. Gradient dilution was performed to obtain the target concentration using DMEM medium. After this, the solution was sterilized through a 0.22 μm filter and dispensed into aliquots.

### 2.4. Cell Culture and Exposure

The cell line used in this study was BV2. The cells were obtained from the Biological and Medical Culture Collection (BNCC), with the accession number BNCC337749. The cell culture medium was utilized as the dispersing solution to prepare different concentrations of CuO-NP-contaminated cell suspension, according to Section 2.1. of the Methods. The cells were cultured in complete medium (containing 10% FBS, 1% penystreptomycin mixture [100 U/mL of penicillin and 100 U/mL of streptomycin], and 89% Dulbecco’s Modified Eagle Medium) at 37 °C in an incubator with 5% CO_2_ saturation humidity. The culture medium was changed daily. Cells were exposed according to the following groups, once they reached 80% confluence: (1) Negative control group (NC): no toxic treatment; (2) Large-particle-size CuO-NPs (L-CuO-NPs): received 5, 10, 15, 20, 25, 30, 35, 40, 45, and 50 μg/mL of large-particle-size CuO-NPs for contamination; (3) Small-particle-size CuO-NPs (small CuO-NPs, S-CuO-NPs): treated with Cu^2+^ with a molar mass of 5, 10, 15, 20, 25, 30, 35, 40, 45, and 50 μg/mL of small-particle-size CuO-NPs; (4) CuO-NPs replaced by CuCl_2_ (Equ group): treated with CuCl_2_ with a molar mass equal to that of CuO-NPs. The molar concentrations of the CuCl_2_ groups were 6.25 × 10^−2^, 0.125, 0.1875, 0.25, 0.3125, 0.375, 0.4375, 0.5, 0.5625, and 0.625 mM, respectively; (5) The mass of Cu^2+^ precipitated from CuO-NPs in cell culture solution substituted by CuCl_2_ (Pre group): treated with CuCl_2_ at the Cu^2+^ concentration previously detected by ICP-MS, which reflected the mass of Cu^2+^ precipitated in the cell culture with CuO-NPs. Each group was set up with three multiple holes and incubated in a CO_2_ incubator for 12 h and 24 h, and all indexes were detected.

### 2.5. Effects of CuO-NPs on BV2 Cell Activity, Determined Using Cell Counting Kit-8 (CCK8) Method

BV2 cells of the logarithmic growth stage were prepared in a cell suspension, and 100 μL of the cell suspension was added into 96-well plates, with each well containing approximately 4 × 10^3^ BV2 cells. The 96-well plates were then cultured in a CO_2_ incubator for 24 h. After culture, the exposure experiment was conducted. After incubation for 12 h and 24 h and after discarding the old medium, a medium containing 10% CCK8 solution (Zinke spectrometry) was added to each cell hole and incubated for 2 h. Absorbance at 450 nm was measured using enzyme markers. The following formula was used: cell relative survival rate (%) = (OD treatment group − OD blank group)/(OD control group − OD blank group) × 100%. The experiment was repeated three times.

### 2.6. Cytoplasmography

An inverted phase contrast microscope (Olympus BX51, Tokyo, Japan) was used to observe the cell changes in the L-CuO-NP, S-CuO-NP, Equ, and Pre groups with 10 m µg/mL of CuO-NPs and corresponding CuCl_2_ concentrations, respectively, after 12 h and 24 h; this was not conducted for the NC group.

### 2.7. Apoptosis Detection Using Flow Cytometry

Cells of the logarithmic growth stage were inoculated with 5 × 10^5^ cells/well into 6-well plates. The NC group cells were not treated with poison, whereas the Pre and S-CuO-NP groups received 5, 10, 20, 30, 40, and 50 μg/mL of CuO-NPs and the corresponding CuCl_2_ concentration for 12 h and 24 h, respectively. The cells were digested and collected with pancreatic enzyme without ethylenediaminetetraacetic acid, washed with phosphate-buffered saline twice, and centrifuged at 1000 rpm for 5 min, and the supernatant was discarded. The apoptosis rate was detected using a double-staining apoptosis kit (Becton Dickinson, Franklin Lakes, NJ, USA). The cells were first resuspended with 200 µL of Annexin V-FITC binding solution, then stained and mixed with 5 µL of Annexin V-FITC and 10 µL of propyl iodide. They were incubated at room temperature (20–25 °C), without light, for 15 min, using using BD FACSCalibur flow cytometer (Becton Dickinson, Franklin Lakes, NJ, USA).

### 2.8. Cell Integrity Detection

Lactate dehydrogenase (LDH) leakage is another objective indicator of cytotoxicity based on membrane integrity. Extracellular LDH levels were measured using the LDH kit (Beyotime Biotech, Shanghai, China). After 12 h and 24 h of full-concentration treatment of BV2 cells in the S-CuO-NPs group, the cell culture medium was collected and centrifuged at 3000 rpm for 10 min, and the supernatant was taken and operated by following the kit instructions. The optical density at 490 nm was measured using the MD SpectraMax M5e.

### 2.9. Identification of TNF-α, IL-1β, and IL-6 Levels Using Enzyme-Linked Immunosorbent Assay (ELISA)

Cells in the NC group were not treated with CuO-NPs, whereas the BV2 cells in the S-CuO-NP group received full concentrations for 12 h and 24 h. The cells were then collected, and the TNF-α, IL-1β, and IL-6 levels in the cells were identified using an ELISA kit (Yunclon, Wuhan, China), following the manufacturer’s instructions. Absorbance was measured at 440 nm using an enzyme marker (model SpectraMax 190 [Molecular Devices, San Jose, CA, USA]).

### 2.10. Cell Oxidative Stress Level Detection and ROS Determination

The NC group was not treated with CuO-NPs, whereas the BV2 cells in the S-CuO-NPs group received a full concentration for 12 h and 24 h, and ROS levels were detected using an ROS kit (Dojindo, Kumamoto, Japan). Intracellular ROS production was measured using a fluorescent probe 2′, 7′ -dichlorofluorescein diacetate (DCF-DA), following the manufacturer’s instructions. Absorbance was measured at 450 nm using the SpectraMax 190 (Molecular Devices, San Jose, CA, USA).

Cells in the NC group were not treated with CuO-NPs, whereas those in the S-CuO-NP group received 5, 10, 20, 30, 40, and 50 μg/mL of CuO-NPs and the corresponding CuCl_2_ concentration for 12 h and 24 h. The superoxide dismutase (SOD), glutathione peroxidase (GSH), and malondialdehyde (MDA) (Wuhan, China) levels were detected using commercial kits, following the kit instructions. Absorbance was measured at 450 nm, utilizing the SpectraMax 190 (Molecular Devices, San Jose, CA, USA).

### 2.11. Western Blot (WB)

The NC group was not treated, whereas the BV2 cells of the S-CuO-NPs group received 5, 10, 20, 30, 40, and 50 μg/mL of CuO-NPs for 24 h, and the cells were collected. WB was used to detect colony-stimulating factor 1 receptor (CSF-1R) and phospholipase C gamma 2 (PLCG-2), extracellular signal-regulated kinase (ERK), nuclear factor erythroid 2-related factor 2 (Nrf2), Fractalkine receptor 1 (CX3CR1), brain-derived neurotrophic factor (BDNF), insulin-like growth factor 1 (IGF-1), B-cell lymphoma/leukemia-2 (Bcl-2), and Bax protein. After fully cracking the cells in an ice bath, they were centrifuged at 12,000 rpm for 5 min (4 °C), and the supernatant was taken. After quantifying the protein concentration using a BCA protein detection kit (Bestbio, Shanghai, China), the cells were denatured by boiling at 95 °C for 10 min. Sodium dodecyl sulfate–polyacrylamide gel electrophoresis separation gel and concentrated gel were prepared, electrophoresis was conducted after labeling was completed, and Tris-buffered saline Tween was used to wash and seal the gel after completing the membrane transfer. Moreover, anti-CSF-1R, P-CSF-1R (1:1000, Proteintech, Wuhan, China), PLCG-2, p-PLCG-2, ERK, p-ERK, Nrf2, p-Nrf2, CX3CR1, BDNF, IGF-1, Bax, and Bcl-2(1:1000, Abcam, Cambridge, UK) were added. Glyceraldehyde 3-phosphate dehydrogenase (1:2000, Abcam, UK) was used for internal argumentation. The cells were incubated at 4 °C overnight. After washing the film, an anti-HRP secondary antibody (1:20,000, Abcam, UK) was added and incubated in a shaker at room temperature for 60 min. Light incubation, image collection, and analyses were performed using ImageJ software (Version 1.53k, National Institutes of Health, Bethesda, MD, USA).

### 2.12. Statistical Analysis

Data were expressed as the mean ± standard deviation. Software was used to conduct a one-way analysis of variance to evaluate the statistical significance of the differences. Tukey’s postmortem test was conducted for multiple comparisons. The statistical significance was set at *p*-values of <0.05.

## 3. Results

### 3.1. CuO-NP Particle Size Characterization

**Figure 1:**
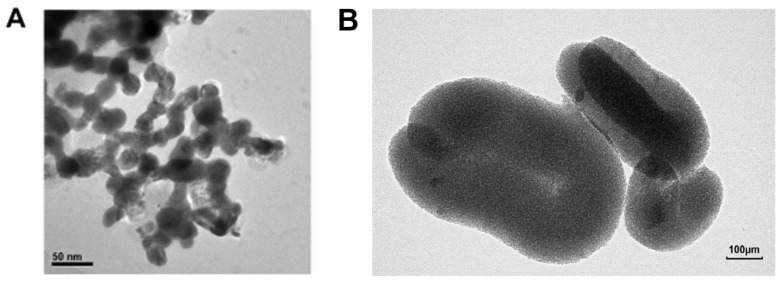
TEM image of CuO-NPs. (**A**) S-CuO-NP granules; (**B**) L-CuO-NP granules.

TEM observations revealed that the CuO-NPs were spherical in shape (Figure 1). The size of the CuO particles with a small particle size was between 20 and 40 nm (Figure 1A); detailed information is shown in Table 1 [39]. The size of the CuO particles with a large particle size was approximately 500 nm (Figure 1B). Their detailed structure and characteristic parameters, which were obtained from the supplier (Sigma-Aldrich, Darmstadt, Germany), are given in Table 1 [39].

### 3.2. Cu^2+^ Level of CuO-NPs Precipitated in Cell Culture Medium

ICP-MS was utilized to analyze the level of Cu^2+^ precipitated in the cell culture solution with different CuO-NP concentrations. The results in Table 2 reveal that the Cu^2+^ concentration accounted for approximately 1/10 of the corresponding CuO-NP concentration. Accordingly, cells in the Pre group were treated with CuCl_2_ at the corresponding concentration determined by ICP-MS to mimic the Cu^2+^ released from CuO-NPs.

### 3.3. Cell Activity

**Figure 2:**
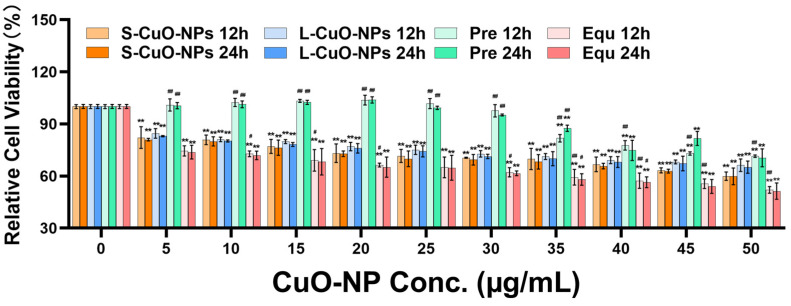
Cell activity measured by CCK8 (*n* = 3). ** *p* < 0.01, compared with control group; # *p* < 0.05, compared with S-CuO-NP group at 12 h and 24 h; ## *p* < 0.01, compared with S-CuO-NP group at 12 h and 24 h. CCK8: Cell Counting Kit-8; S-CuO-NP: small-size copper oxide nanoparticle.

Compared with the control group, the cell activity of the S-CuO-NP, L-CuO-NP, and Equ groups was significantly decreased. The Pre group demonstrated a downward trend, and the concentration of 35–50 μg/mL was significantly decreased. The cell activity decreased dose-dependently. Compared with the 12 h treatment, the cell proliferation activity decreased after the 24 h treatment. The toxicity of all groups increased, and was highest in the Equ group, followed by the S-CuO-NP, L-CuO-NP, and Pre groups, respectively (Figure 2).

### 3.4. Cell Morphology Observation

**Figure 3:**
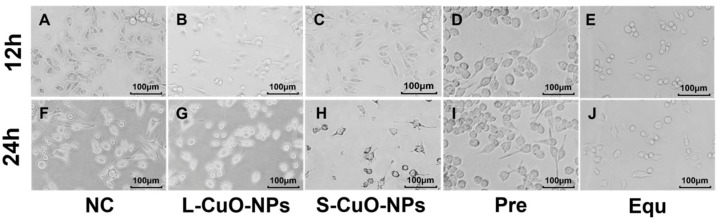
Images from 40× inverted light microscope for BV2 cells. (**A**) Image of control cells at 12 h. (**B**) Image of L-CuO-NP-contaminated cells at 12 h. (**C**) Image of S-CuO-NP-contaminated cells at 12 h. (**D**) Image of cells in Cu^2+^ group with precipitation of CuO-NPs at 12 h. (**E**) Image of cells in Cu^2+^ group with equal molar mass and CuO-NPs at 12 h. (**F**) Image of cells in control group at 24 h. (**G**) Image of L-CuO-NP-contaminated cells at 24 h. (**H**) Image of S-CuO-NP-contaminated cells at 24 h. (**I**) Image of cells in Cu^2+^ group with CuO-NP precipitation at 24 h. (**J**) Image of cells in Cu^2+^ group with equal molar mass and CuO-NPs at 24 h. CuO-NP: copper oxide nanoparticles; L-CuO-NP: large-size CuO-NP; S-CuO-NP: small-size CuO-NP; Cu^2+^: CuO-NP and its copper ion.

The morphology of microglia changes significantly when they are damaged or activated. First, the cells shift from a resting state to an active state, with the cells becoming larger and rounder, exhibiting an amoeba-like appearance. Thickening and shortening of the dendrimers and an increase in the density of the receptors on the cell surface accompany this morphological change. The morphological results (Figure 3) reveal that BV2 cells that were inoculated and cultured normally were semi-floating cells with typical small branching fusiform morphology. Hence, microglia in the resting state were thin, their cell bodies were small, round, or oval, and the synapses were slender. Cell growth was slow at 12 h, the number of cells increased exponentially after 24 h, and contact inhibition appeared. After treating the cells with 10 μg/mL of CuO-NPs and the corresponding CuCl_2_ concentration, the branches of individual small branching cells were shortened and enlarged, and demonstrated an amoeba-like appearance. Some BV2 cells lost their original form and became cramped, and the number of cells was reduced relative to the number of normal incubated cells. The morphological damage toxicity was highest in the Equ group, followed by the S-CuO-NPs, L-CuO-NPs, and Pre groups, respectively. The increase was time-dependent, which was consistent with the changing trend of living cell activity.

### 3.5. Apoptosis

**Figure 4:**
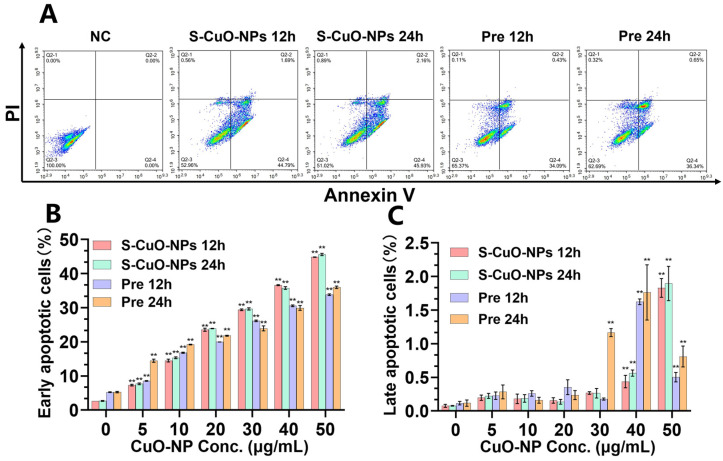
Apoptosis was detected using flow cytometry. (**A**) Result of 50 μg/mL FCM detection. (**B**) Proportion of early apoptotic cells in different groups with various concentrations (*n* = 3). (**C**) Proportion of late apoptotic cells in different groups with various concentrations (*n* = 3). ** *p* < 0.01, compared with the control group (group at 0 h).

Compared with the control group, the S-CuO-NP and Pre group BV2 cells demonstrated significant early apoptosis after treatment for 12 h and 24 h (Figure 4B). The levels of late apoptotic cells in the S-CuO-NP and Pre group BV2 cells demonstrated an increasing trend, being significantly elevated at the concentration of 40–50 μg/mL (Figure 4C). At the same concentration and time, the degree damage caused by S-CuO-NPs to BV2 cells was greater than that observed for cells in the Pre group. Moreover, the damage to BV2 cells at 24 h was greater than that at 12 h. The apoptosis results further confirmed that the toxicity of CuO-NPs was greater than that in the Pre group. Therefore, S-CuO-NPs were then used to further investigate the mechanism of CuO-NP damage to microglia.

### 3.6. Extracellular LDH Levels

**Figure 5:**
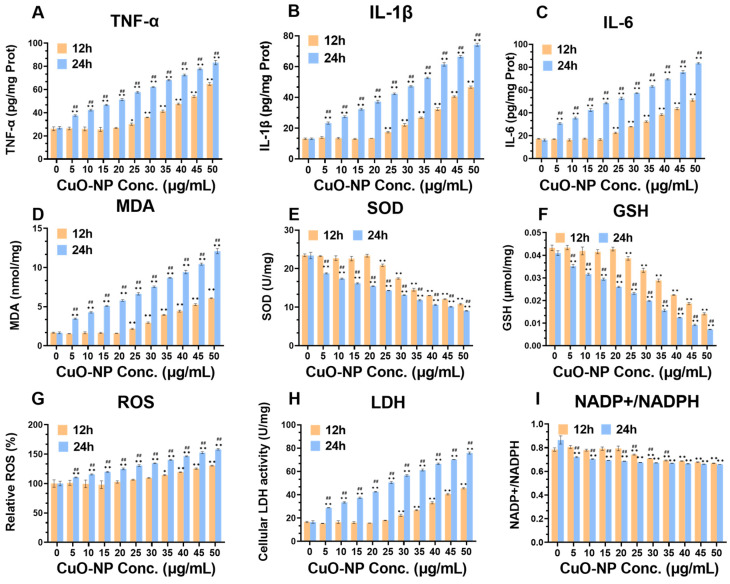
Results of cellular inflammation and oxidative damage. (**A**) Tumor necrosis factor-α (TNF-α) (*n* = 3); (**B**) interleukin-1β (IL-1β) (*n* = 3); (**C**) interleukin-6 (IL-6) (*n* = 3); (**D**) malondialdehyde (MDA) (*n* = 3); (**E**) superoxide dismutase (SOD) (n = 3); (**F**) glutathione (GSH) (n = 3); (**G**) reactive oxygen species (ROS) (*n* = 3); (**H**) lactate dehydrogenase (LDH) (*n* = 3); (**I**) nicotinamide adenine dinucleotide phosphate/reduced nicotinamide adenine dinucleotide phosphate (NADP/NADPH) (*n* = 3). * *p* < 0.05, compared with control group (group at 0 h; ** *p* < 0.01, compared with control group (0 h group); ## *p* < 0.01, compared with control group (S-CuO-NPs group at 12 h and 24 h).

The LDH leakage rate was significantly increased with elevated CuO-NP concentration. The LDH leakage rate of BV2 cells significantly increased in the 24 h group compared to in the 12 h group (FIG. 5H), indicating improved membrane permeability and further revealing that BV2 cells were damaged by CuO-NP exposure.

### 3.7. Cellular Oxidative Stress and Inflammation Levels

The results of inflammatory level analysis (Figure 5A–C) revealed that TNF-α, IL-1β, and IL-6 levels in BV2 cells were significantly increased after 24 h of treatment with S-CuO-NPs compared to the control, indicating a dose-dependent elevation. TNF-α, IL-1β, and IL-6 levels in the cells tended to increase after 12 h of treatment, which was significant at concentrations of 25–50 μg/mL, and shows that they were affected in a time-dependent manner. Meanwhile, compared with the control group, the intracellular ROS and MDA levels increased significantly after 24 h of treatment, and the levels gradually increased with elevated CuO-NP concentration, whereas the SOD activity and GSH levels significantly and gradually decreased with elevated CuO-NP concentration, in a time-dependent manner (Figure 5D–G). The ratio of NADP+/NADPH increased with an increase in CuO-NP concentration in a time-dependent manner (Figure 5I), and the difference was statistically significant (*p* < 0.05). These results indicate that BV2 cell damage caused by CuO-NP exposure is related to the inflammatory response and oxidative stress.

### 3.8. CuO-NPs Cause Functional Damage to BV2 Cells and Promote BV2 Apoptosis Through CSF-1R/PLCγ2/ERK/Nrf2 Signaling Pathway and Its Effect on Level of Functional Damage Protein and Damage Mechanism

**Figure 6:**
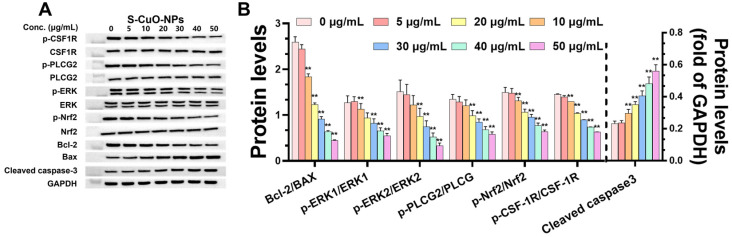
Effect of CuO-NPs on apoptotic protein levels in BV2 cells. (**A**) Apoptotic pathway WB grayscale map. (**B**) Expression levels and cleaved caspase-3 ratio of p-CSF-1R/CSF-1R, p-PLCγ2/PLCγ2, p-ERK/ERK, p-Nrf2/Nrf2, Bcl-2/Bax, and cleaved caspase-3 (n = 3). ** *p* < 0.01, compared with control group (group at 0 h).

To further investigate the mechanism of neuronal injury induced by CuO-NPs, the protein expression related to apoptosis was detected after 24 h of treatment with S-CuO-NPs. The results revealed that compared with the control group, the apoptotic proteins p-CSF-1R/CSF-1R, p-Nrf2/Nrf2, and Bcl-2/Bax, associated with neuronal injury and degenerative diseases, were significantly decreased, and cleaved caspase-3 expression was significantly increased. Further, p-PLCγ2/PLCγ2 and p-ERK/ERK were significantly decreased at CuO-NP concentrations of 20–50 μg/mL, in a dose-dependent manner (Figure 6A,B). CuO-NPs induced oxidative stress and inflammation in BV2 cells through the CSF-1R/PLCγ2/ERK/Nrf2 pathway, causing apoptosis of BV2 cells.

**Figure 7:**
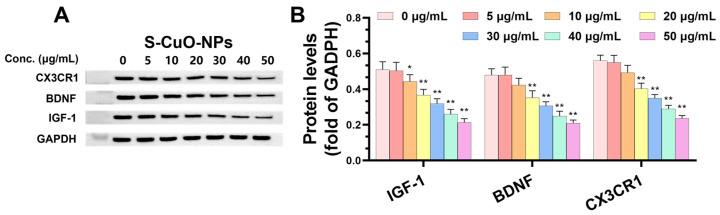
Effect of CuO-NPs on neurofunctional protein levels in BV2 cells. (**A**) Neural function WB grayscale map; (**B**) expression levels of CX3CR1, CSF-1R, and IGF-1 (*n* = 3). * *p* < 0.05, compared with control group (group at 0 h); ** *p* < 0.01, compared with control group (group at 0 h).

The expression of CX3CR1 was significantly decreased in the exposed group compared to in the control group, and BDNF and IGF-1 expression levels were significantly decreased when the CuO-NP concentration was 20–50 μg/mL, in an efficiency–time-dependent manner (Figure 7A,B). These results indicate that CuO-NPs reduced the protective and repairing ability of BV2 cells in the nervous system.

## 4. Discussion

In this study, we first conducted a series of experiments to explore the toxicity of different substances related to CuO-NPs. Given that the potential toxicity differences between CuO-NPs and copper ions were not fully clear, we designed the initial experimental groups. We aimed to compare the toxicity of CuO-NPs with different particle sizes (S-CuO-NPs, L-CuO-NPs), Cu^2+^ of an equal molar weight (Equ group), and CuCl_2_ with an equal molar weight of Cu^2+^ separated from CuO-NPs (Pre group). After co-culturing these substances with BV2 microglia for 12 and 24h, we obtained results regarding cell activity, morphology, and apoptosis. These results clearly showed the toxicity order of Equ Group > S-CuO-NPs > L-CuO-NPs > Pre group, and showed that the toxicity increased in a time- and dose-dependent manner.

Based on these findings, we then focused on the mechanism of damage cause by S-CuO-NPs to cells. CuO-NPs caused morphological changes in the microglia, increased oxidative stress and inflammation levels, and destroyed the integrity of the cell membrane, resulting in cell apoptosis and decreased cell activity.

The toxicity mechanism of CuO-NPs has two main aspects [40,41]. First, the released Cu^2+^ exerts ionic toxicity in organisms. CuO-NPs precipitate a large amount of Cu^2+^ in the cell media [42], causing cell damage. Second, CuO-NPs directly cause oxidative stress and physical damage to body cells. When a small number of CuO-NPs enter the cell, a large number of ROS can be produced [43], which affects mitochondrial respiration and apoptosis, causing REDOX imbalance in the cell and peroxidation in the cell membrane [44], resulting in cell damage. Further, CuO-NPs move to the nucleus through direct physical damage to the nuclear pore or membrane, causing cell damage [45]. In this study, the damage and mechanism of BV2 microglia induced by CuO-NPs with different particle sizes, Cu^2+^ with equal molar mass, and CuO-NPs deposited in the cell culture solution for 12 h and 24 h were investigated. The results of cell activity and morphology revealed the highest toxicity in the Equ group, followed by the S-CuO-NP, L-CuO-NP, and Pre groups, respectively. The toxic effect of the CuO-NPs itself was higher than that of the Cu^2+^ released by CuO-NPs, and the toxicity of the different treatments was consistent with cell activity and morphology results. In particular, we revealed that copper ions with equal molar mass were more toxic than CuO-NPs themselves, as also reported in previous studies [46]. Building on these in vitro findings, it is worth noting that in vivo copper homeostasis is strictly regulated by transporters such as ATP7A/B and metallothioneins. The concentration of free Cu^2+^ is usually maintained at the nanomolar level, which is much lower than the concentration of Cu^2+^ released by CuO-NPs in in vitro experiments [47,48]. Different cells exhibit diverse sensitivities and response patterns to nanoparticles. Specifically, for CuO-NPs (copper oxide nanoparticles), toxicity varies with particle size [49]. In our study, we chose two distinct CuO-NP size ranges: 20–40 nm and 500 nm. The 20–40 nm CuO-NPs, being smaller, likely have a larger specific surface area than the 500 nm ones. This surface area difference implies more intense cell interactions for the 20–40 nm NPs. They may penetrate cells more easily, causing greater ROS production and more substantial disruption to cellular functions. Conversely, the larger 500 nm CuO-NPs may interact with cells less effectively, potentially having a lower impact on cellular processes and a different toxicity profile. Understanding this size-related toxicity difference is crucial in our research, as it can offer insights into cellular responses to variously sized CuO-NPs.

BV2 cells, a subtype of microglial cells within the immune cell family, may possess a certain level of resistance to CuO-NPs. As immune cells, they are endowed with self-defense and regulatory mechanisms. These innate capabilities enable BV2 cells to potentially sustain their metabolic activity through self-regulation, even when subjected to relatively high doses of CuO-NPs [49]. Notably, compared to ordinary cells, BV2 cells might exhibit a higher survival rate under identical exposure circumstances [37]. This could be attributed to their unique immune-associated features, which equip them to better withstand the stress imposed by CuO-NPs. Nevertheless, despite this demonstrated resilience, a more in-depth exploration of their response mechanisms to nanoparticles, considering their distinct characteristics, remains imperative. Such knowledge is vital for a comprehensive assessment of the influence of CuO-NPs on the immune-related functions of BV2 cells and their role in the overall cellular response to nanoparticle exposure.

To further investigate the mechanism of CuO-NP damage to microglia, we treated BV2 cells with S-CuO-NPs for 24 h and examined the pathway of apoptosis induced by CuO-NPs. Activation of the CSF-1R/PLCγ2/ERK/Nrf2 pathway may be associated with inflammatory and neurodegenerative diseases. CSF-1R plays a crucial role in neurodegenerative diseases [50]. In the CNS, CSF-1R is mainly expressed in microglia, and plays an important role in the growth, development, cell function, and neurophenotype of neurons and microglia [51,52,53]. CSF-1R deficiency will affect microglia development and maturation and homeostasis maintenance, causing neuroinflammation [54], thereby reducing the nutritional support of microglia to neurons and the regulation of neurogenesis [55], and inhibiting the activity of microglia. Moreover, mutations in CSF-1R cause neurodegenerative and inflammatory diseases [56,57]. PLCγ2 is located downstream of CSF-1R and is a component of CSF1-induced microglia differentiation after triggering an immune response. PLCγ2 is a membrane-associated enzyme that plays a crucial role in cell surface receptor signaling. Previous studies have revealed that PLCγ2 expression increases dependently with AD progression, and PLCγ2 is highly expressed in patch-induced brain microglia [58]. PLCγ2 is the primary effector protein of CSF1/CSF-1R, which participates in mediating the biological functions of CSF1/CSF-1R in neuronal apoptosis and neuroinflammation [59], and plays the biological function of alleviating cellular inflammation, oxidative stress, and apoptosis through different downstream molecules [60]. The activation products of PLCγ2 activate the ERK signaling pathway, and studies have demonstrated that PLCγ2 promotes hepatocyte proliferation through the ERK pathway [61]. ERK1/2 is highly expressed, and plays different biological functions in various cells. It is involved in different cell signal transduction pathways, and mainly regulates cell proliferation, differentiation, apoptosis, and stress response. As the upstream pathway of Nrf2 [62], ERK activation promotes the nuclear translocation of Nrf2, thereby improving the antioxidant capacity of Nrf2 [63,64]. When cells are subjected to external stimuli (e.g., growth factors, cytokines), ERK is activated, which then regulates the phosphorylation of downstream target proteins [65], affecting the physiological functions of cells. Previous studies have revealed the involvement of Nrf2 in neurobiological activities or functions such as memory and cognitive impairment, oxidative stress, nerve damage, and apoptosis [66,67,68,69,70].

ERK phosphorylation regulates the direction of cell differentiation, as well as inhibiting apoptosis during cell survival. Nrf2 is an important transcription factor that is related to protecting target gene expression, alleviating oxidative stress, and maintaining intracellular environmental homeostasis. A previous study reported that the Nrf2 signaling pathway activation changes the degree of oxidative damage induced by Aβ in SH-SY5Y cells [71]. The proapoptotic member Bax, the antiapoptotic member Bcl-2, and downstream cleaved caspase-3 play a key regulatory role in apoptosis, and are frequently used for apoptosis assessment [72]. An increase in the Bax/Bcl-2 ratio can upregulate caspase-3 to induce apoptosis, and caspase-3 upregulation and related pathway activation are considered the main pathways of neuronal apoptosis [44]. In this study, we revealed that CuO-NPs reduced the expression ratio of p-CSF-1R/CSF-1R, p-PLCγ2/PLCγ2, p-ERK1/2/ERK1/2, and p-Nrf2/Nrf2 proteins in BV2 cells in a concentration-dependent manner [43]. Concurrently, the Bax/Bcl-2 ratio of BV2 and the cleaved caspase-3 protein level were significantly upregulated. These results indicate that CuO-NPs may cause the homeostatic imbalance and apoptosis of nerve cells and neurons, including BV2 microglia, through the CSF-1R pathway, and participate in the process of cellular oxidative stress, inflammation, and apoptosis through PLCγ2. CuO-NP treatment reduced ERK1/2 phosphorylation and Nrf2 nuclear translocation by inhibiting ERK signaling in BV2 cells, thereby destroying intracellular homeostasis. In conclusion, our study revealed that CuO-NPs induced oxidative stress and inflammation in BV2 cells through the CSF-1R/PLCγ2/ERK/Nrf2 pathway, resulting in cell apoptosis.

Similarly, CX3CR1, BDNF, and IGF1, which are associated with microglia function, were also downregulated at different levels. IGF1, a microglia-derived neurotrophic factor, promotes neuronal survival and inhibits apoptosis [55]. This study confirmed that CuO-NPs induced a concentration-dependent decrease in IGF-1 in BV2 microglia, indicating the protective effect of microglia on neurons. CX3CR1 is a fractalkine receptor and a common marker of microglial expression, which is mainly expressed in monocytes, macrophages, dendritic cells, T cells, and natural killer cells [73]. In the CNS, CX3CR1, which is specifically expressed by microglia and is closely related to microglia, can be utilized as a link or target to inhibit microglia activation [74]. The downregulation or loss of the expression level of CX3CR1 and other receptors improves phagocytosis of microglia and increases the release of inflammatory cytokines [75], thereby causing a toxic effect on neurons and triggering neurological toxicity. Further, CX3CR1 signaling dysregulation inhibits synaptic pruning, resulting in associated cognitive dysfunction [76]. In this study, CuO-NPs caused CX3CR1 signal dysregulation in BV2 microglia, with a concentration-dependent decrease, which confirms that CuO-NPs may cause neuronal damage by triggering the imbalance of microglia homeostasis and promoting neurodegenerative disease occurrence. BDNF is primarily expressed in the CNS, and plays a crucial role in cell survival and neuronal differentiation [77]. When neurons are damaged by hypoxia, excitotoxicity, etc., BDNF activates the intracellular antiapoptotic mechanism and increases the survival probability of neurons. Concurrently, BDNF increases the plasticity of the cortical dendritic spines, and synaptic loss is considered an accurate indicator of cognitive decline in AD [78]. This study revealed BDNF downregulation in a concentration-dependent manner after BV2 cells were exposed to CuO-NPs, indicating that CuO-NPs affect neuronal differentiation by inhibiting microglia-derived BDNF. They exert a catalytic effect on the occurrence of neurodegenerative diseases, which may be caused by them affecting BV2 cells at the synaptic level.

## 5. Conclusions

Our study confirms that the toxic effect of CuO-NPs on microglia primarily originates from the nanoparticle itself; a small amount comes from the precipitated Cu^2+^, and the toxicity to cells was successively equal to the molar amount of Cu^2+^, S-CuO-NPs, L-CuO-NPs, and the precipitated amount of Cu^2+^, respectively. CuO-NPs induced oxidative stress and inflammation in BV2 cells through the CSF-1R/PLCγ2/ERK/Nrf2 pathway, causing cell apoptosis. Concurrently, this study revealed that CX3CR1, BDNF, and IGF1 in BV2 cells were downregulated to varying degrees after CuO-NP exposure, indicating that CuO-NPs damaged the neural function of microglia. The ability of microglia to support peripheral neurons to maintain survival, promote the growth and differentiation of neurons, and fight against apoptosis is reduced by exposure to CuO-NPs, making neurons vulnerable to further damage, and resulting in nervous system dysfunction and the occurrence and development of neurodegenerative diseases.

## Figures and Tables

**Table 1:** Characteristics of Cuo-NPs.

Size (nm)	Density (g/m^3^)	SSA (m^2^/g)	Crystalline Structure	Shape	Composition
500	6.3–6.49	10.1	Cubic/tetragonal	Nearly spherical	99.9%
20–40	6.3–6.49	29	Monoclinic	Sphere	99.9%

**Table 2:** Precipitation degree of nanometer copper oxide in complete medium for 24 h (n = 3).

Concentration of S-CuO-NPs (μg/mL)	Concentration of Cu^2+^ (μg/mL)	CuCl_2_ with Molar Mass Equal to Cu^2^ (mM)
0	0 ± 0	0
0.5	0.02 ± 0.03	3.15 × 10^−4^
1	0.06 ± 0.01	9.44 × 10^−4^
1.5	0.09 ± 0.03	1.42 × 10^−3^
2	0.1 ± 0.03	1.57 × 10^−3^
2.5	0.11 ± 0.04	1.73 × 10^−3^
3	0.17 ± 0.04	2.68 × 10^−3^
3.5	0.13 ± 0.02	2.05 × 10^−3^
4	0.35 ± 0.03	5.51 × 10^−3^
4.5	0.33 ± 0.03	5.19 × 10^−3^
5	0.38 ± 0.03	5.98 × 10^−3^
10	0.86 ± 0.04	1.35 × 10^−2^
15	1.26 ± 0.05	1.98 × 10^−2^
20	1.65 ± 0.09	2.60 × 10^−2^
25	2.22 ± 0.04	3.49 × 10^−2^
30	2.7 ± 0.15	4.25 × 10^−2^
35	3.24 ± 0.15	5.10 × 10^−2^
40	3.64 ± 0.07	5.73 × 10^−2^
45	4.19 ± 0.11	6.59 × 10^−2^
50	4.72 ± 0.09	7.43 × 10^−2^

## Data Availability

The data that support the findings of this study are available from the corresponding author upon reasonable request.

## References

[1] Angelé-Martínez C., Nguyen K.V., Ameer F.S., Anker J.N., Brumaghim J.L. (2017). Reactive oxygen species generation by copper(II) oxide nanoparticles determined by DNA damage assays and EPR spectroscopy. Nanotoxicology.

[2] Anderson A.J., McLean J.E., Jacobson A.R., Britt D.W. (2018). CuO and ZnO Nanoparticles Modify Interkingdom Cell Signaling Processes Relevant to Crop Production. J. Agric. Food. Chem..

[3] Pierzynowska K., Kamińska T., Węgrzyn G. (2020). One drug to treat many diseases: Unlocking the economic trap of rare diseases. Metab. Brain Dis..

[4] Gao C., Jiang J., Tan Y., Chen S. (2023). Microglia in neurodegenerative diseases: Mechanism and potential therapeutic targets. Signal Transduct. Target. Ther..

[5] Sarlus H., Heneka M.T. (2017). Microglia in Alzheimer’s disease. J. Clin. Investig..

[6] Arcuri C., Mecca C., Bianchi R., Giambanco I., Donato R. (2017). The Pathophysiological Role of Microglia in Dynamic Surveillance, Phagocytosis and Structural Remodeling of the Developing CNS. Front. Mol. Neurosci..

[7] Liang X.Y., Zhang Z.Q., Zhang Y., Liu Y., Wang X. (2018). Effects of zinc oxide nanoparticle on expression of inflammatory factors and phosphorylation of p38MAPK in BV2 microglia. J. Environ. Occup. Med..

[8] Marmiroli M., Pagano L., Rossi R., De La Torre-Roche R., Lepore G.O., Ruotolo R., Gariani G., Bonanni V., Pollastri S., Puri A. (2021). Copper Oxide Nanomaterial Fate in Plant Tissue: Nanoscale Impacts on Reproductive Tissues. Environ. Sci. Technol..

[9] Muzio L., Viotti A., Martino G. (2021). Microglia in Neuroinflammation and Neurodegeneration: From Understanding to Therapy. Front. Neurosci..

[10] Kinney J.W., Bemiller S.M., Murtishaw A.S., Leisgang A.M., Salazar A.M., Lamb B.T. (2018). Inflammation as a central mechanism in Alzheimer’s disease. Alzheimer’s Dement..

[11] Badanjak K., Fixemer S., Smajić S., Skupin A., Grünewald A. (2021). The Contribution of Microglia to Neuroinflammation in Parkinson’s Disease. Int. J. Mol. Sci..

[12] McGeer P.L., McGeer E.G. (2002). Inflammatory processes in amyotrophic lateral sclerosis. Muscle Nerve.

[13] Duan W., Wang H., Fan Q., Chen L., Huang H., Ran H. (2018). Cystatin F involvement in adenosine A2A receptor-mediated neuroinflammation in BV2 microglial cells. Sci. Rep..

[14] Biswas K. (2023). Microglia mediated neuroinflammation in neurodegenerative diseases: A review on the cell signaling pathways involved in microglial activation. J. Neuroimmunol..

[15] Liu Z., Yao X., Jiang W., Li W., Zhu S., Liao C., Zou L., Ding R., Chen J. (2020). Advanced oxidation protein products induce microglia-mediated neuroinflammation via MAPKs-NF-κB signaling pathway and pyroptosis after secondary spinal cord injury. J. Neuroinflamm..

[16] Pramanik S., Devi M.H., Chakrabarty S., Paylar B., Pradhan A., Thaker M., Ayyadhury S., Manavalan A., Olsson P.-E., Pramanik G. (2024). Microglia signaling in health and disease—Implications in sex-specific brain development and plasticity. Neurosci. Biobehav. Rev..

[17] Bulua A.C., Simon A., Maddipati R., Pelletier M., Park H., Kim K.Y., Sack M.N., Kastner D.L., Siegel R.M. (2011). Mitochondrial reactive oxygen species promote production of proinflammatory cytokines and are elevated in TNFR1-associated periodic syndrome (TRAPS). J. Exp. Med..

[18] Feng-Shiun S., Randall L.W. (2007). Manipulation of Microglial Activation as a Therapeutic Strategy in Alzheimers Disease. Curr. Med. Chem..

[19] Kim Y.S., Joh T.H. (2006). Microglia, major player in the brain inflammation: Their roles in the pathogenesis of Parkinson’s disease. Exp. Mol. Med..

[20] Karlsson H.L., Cronholm P., Gustafsson J., Möller L. (2008). Copper oxide nanoparticles are highly toxic: A comparison between metal oxide nanoparticles and carbon nanotubes. Chem. Res. Toxicol..

[21] Song M.F., Li Y.S., Kasai H., Kawai K. (2012). Metal nanoparticle-induced micronuclei and oxidative DNA damage in mice. J. Clin. Biochem. Nutr..

[22] Doudi M., Setorki M. (2014). Acute effect of nano-copper on liver tissue and function in rat. Nanomed. J..

[23] Mohammadyari A., Razavipour S.T., Mohammadbeigi M., Negahdary M., Ajdary M. (2014). Exploring vivo toxicity assessment of copper oxide nanoparticle in Wistar rats. J. Biol. Today’s World.

[24] Ramyadevi J., Jeyasubramanian K., Marikani A., Rajakumar G., Rahuman A.A. (2012). Synthesis and antimicrobial activity of copper nanoparticles. Mater. Lett..

[25] Cuillel M., Chevallet M., Charbonnier P., Fauquant C., Pignot-Paintrand I., Arnaud J., Cassio D., Michaud-Soret I., Mintz E. (2014). Interference of CuO nanoparticles with metal homeostasis in hepatocytes under sub-toxic conditions. Nanoscale.

[26] Bopp S.K., Abicht H.K., Knauer K. (2008). Copper-induced oxidative stress in rainbow trout gill cells. Aquat. Toxicol..

[27] Sutton H.C., Winterbourn C.C. (1989). On the participation of higher oxidation states of iron and copper in fenton reactions. Free Radical Biol. Med..

[28] Lee I.C., Ko J.W., Park S.H., Lim J.O., Shin I.S., Moon C., Kim S.H., Heo J.D., Kim J.C. (2016). Comparative toxicity and biodistribution of copper nanoparticles and cupric ions in rats. Int. J. Nanomedicine.

[29] Ganganelli I., Galatro A., Gergoff Grozeff G.E., Bartoli C.G., Senn M.E., Ziogas V., Corpas F.J. (2024). 3-Reactive oxygen species (ROS): Chemistry and role in plant physiology. Oxygen, Nitrogen and Sulfur Species in Post-Harvest Physiology of Horticultural Crops.

[30] Wang L., Huang X., Sun W., Too H.Z., Laserna A.K.C., Li S.F.Y. (2020). A global metabolomic insight into the oxidative stress and membrane damage of copper oxide nanoparticles and microparticles on microalga Chlorella vulgaris. Environ. Pollut..

[31] Jiang M., Tao X., Pang Y., Qin Z., Song E., Song Y. (2024). Copper oxide nanoparticles induce cuproptosis and ferroptosis through mitochondrial concatenation. Environ. Sci. Nano.

[32] Jiang Y.W., Gao G., Jia H.R., Zhang X., Zhao J., Ma N., Liu J.B., Liu P., Wu F.G. (2019). Copper Oxide Nanoparticles Induce Enhanced Radiosensitizing Effect via Destructive Autophagy. ACS Biomater. Sci. Eng..

[33] Cheng W., Zhang W., Xia X., Zhang J., Wang M., Li Y., Li X., Zheng Y., Liu J., Zhang R. (2023). The domino effect in inhaled carbon black nanoparticles triggers bloodbrain barrier disruption via altering circulatory inflammation. Nano Today.

[34] Che X., Ding R., Li Y., Zhang Z., Gao H., Wang W. (2018). Mechanism of long-term toxicity of CuO NPs to microalgae. Nanotoxicology.

[35] Tatsi K., Shaw B.J., Hutchinson T.H., Handy R.D. (2018). Copper accumulation and toxicity in earthworms exposed to CuO nanomaterials: Effects of particle coating and soil ageing. Ecotoxicol. Environ. Saf..

[36] Yue L., Zhao J., Yu X., Lv K., Wang Z., Xing B. (2018). Interaction of CuO nanoparticles with duckweed (*Lemna minor*. L): Uptake, distribution and ROS production sites. Environ. Pollut..

[37] Farshori N.N., Siddiqui M.A., Al-Oqail M.M., Al-Sheddi E.S., Al-Massarani S.M., Ahamed M., Ahmad J., Al-Khedhairy A.A. (2022). Copper Oxide Nanoparticles Exhibit Cell Death Through Oxidative Stress Responses in Human Airway Epithelial Cells: A Mechanistic Study. Biol. Trace Elem. Res..

[38] Sajjad H., Sajjad A., Haya R.T., Khan M.M., Zia M. (2023). Copper oxide nanoparticles: In vitro and in vivo toxicity, mechanisms of action and factors influencing their toxicology. Comp. Biochem. Physiol. C Toxicol. Pharmacol..

[39] Liu H., Lai W., Liu X., Yang H., Fang Y., Tian L., Li K., Nie H., Zhang W., Shi Y. (2021). Exposure to copper oxide nanoparticles triggers oxidative stress and endoplasmic reticulum (ER)-stress induced toxicology and apoptosis in male rat liver and BRL-3A cell. J. Hazard. Mater..

[40] Souza M.R., Mazaro-Costa R., Rocha T.L. (2021). Can nanomaterials induce reproductive toxicity in male mammals? A historical and critical review. Sci. Total Environ..

[41] Hou J., Wang X., Hayat T., Wang X. (2017). Ecotoxicological effects and mechanism of CuO nanoparticles to individual organisms. Environ. Pollut..

[42] Gunawan C., Teoh W.Y., Marquis C.P., Amal R. (2011). Cytotoxic Origin of Copper(II) Oxide Nanoparticles: Comparative Studies with Micron-Sized Particles, Leachate, and Metal Salts. ACS Nano.

[43] Dudev T., Lim C. (2008). Metal binding affinity and selectivity in metalloproteins: Insights from computational studies. Annu. Rev. Biophys..

[44] Xia T., Kovochich M., Liong M., Mädler L., Gilbert B., Shi H., Yeh J.I., Zink J.I., Nel A.E. (2008). Comparison of the Mechanism of Toxicity of Zinc Oxide and Cerium Oxide Nanoparticles Based on Dissolution and Oxidative Stress Properties. ACS Nano.

[45] Wang Y., Aker W.G., Hwang H.-m., Yedjou C.G., Yu H., Tchounwou P.B. (2011). A study of the mechanism of in vitro cytotoxicity of metal oxide nanoparticles using catfish primary hepatocytes and human HepG2 cells. Sci. Total Environ..

[46] Siddiqui S., Goddard R.H., Bielmyer-Fraser G.K. (2015). Comparative effects of dissolved copper and copper oxide nanoparticle exposure to the sea anemone, Exaiptasia pallida. Aquat. Toxicol..

[47] Singla A., Chen Q., Suzuki K., Song J., Fedoseienko A., Wijers M., Lopez A., Billadeau D.D., van de Sluis B., Burstein E. (2021). Regulation of murine copper homeostasis by members of the COMMD protein family. Dis. Model. Mech..

[48] Prohaska J.R. (2008). Role of copper transporters in copper homeostasis. Am. J. Clin. Nutr..

[49] Wongrakpanich A., Mudunkotuwa I.A., Geary S.M., Morris A.S., Mapuskar K.A., Spitz D.R., Grassian V.H., Salem A.K. (2016). Size-dependent cytotoxicity of copper oxide nanoparticles in lung epithelial cells. Environ. Sci. Nano.

[50] Han J., Chitu V., Stanley E.R., Wszolek Z.K., Karrenbauer V.D., Harris R.A. (2022). Inhibition of colony stimulating factor-1 receptor (CSF-1R) as a potential therapeutic strategy for neurodegenerative diseases: Opportunities and challenges. Cell Mol. Life Sci..

[51] Tarale P., Alam M.M. (2022). Colony-stimulating factor 1 receptor signaling in the central nervous system and the potential of its pharmacological inhibitors to halt the progression of neurological disorders. Inflammopharmacology.

[52] Ginhoux F., Greter M., Leboeuf M., Nandi S., See P., Gokhan S., Mehler M.F., Conway S.J., Ng L.G., Stanley E.R. (2010). Fate mapping analysis reveals that adult microglia derive from primitive macrophages. Science.

[53] Erblich B., Zhu L., Etgen A.M., Dobrenis K., Pollard J.W. (2011). Absence of colony stimulation factor-1 receptor results in loss of microglia, disrupted brain development and olfactory deficits. PLoS ONE.

[54] Adams R.C., Carter-Cusack D., Llanes G.T., Hunter C.R., Vinnakota J.M., Ruitenberg M.J., Vukovic J., Bertolino P., Chand K.K., Wixey J.A. (2024). CSF1R inhibition promotes neuroinflammation and behavioral deficits during graft-versus-host disease in mice. Blood.

[55] Arnò B., Grassivaro F., Rossi C., Bergamaschi A., Castiglioni V., Furlan R., Greter M., Favaro R., Comi G., Becher B. (2014). Neural progenitor cells orchestrate microglia migration and positioning into the developing cortex. Nat. Commun..

[56] Mickeviciute G.C., Valiuskyte M., Plattén M., Wszolek Z.K., Andersen O., Danylaité Karrenbauer V., Ineichen B.V., Granberg T. (2022). Neuroimaging phenotypes of CSF1R-related leukoencephalopathy: Systematic review, meta-analysis, and imaging recommendations. J. Intern. Med..

[57] Konno T., Yoshida K., Mizuno T., Kawarai T., Tada M., Nozaki H., Ikeda S.I., Nishizawa M., Onodera O., Wszolek Z.K. (2017). Clinical and genetic characterization of adult-onset leukoencephalopathy with axonal spheroids and pigmented glia associated with CSF1R mutation. Eur. J. Neurol..

[58] Tsai A.P., Dong C., Lin P.B.-C., Messenger E.J., Casali B.T., Moutinho M., Liu Y., Oblak A.L., Lamb B.T., Landreth G.E. (2022). PLCG2 is associated with the inflammatory response and is induced by amyloid plaques in Alzheimer’s disease. Genome Med..

[59] Li K., Ran B., Wang Y., Liu L., Li W. (2022). PLCγ2 impacts microglia-related effectors revealing variants and pathways important in Alzheimer’s disease. Front. Cell Dev. Biol..

[60] Obba S., Hizir Z., Boyer L., Selimoglu-Buet D., Pfeifer A., Michel G., Hamouda M.A., Gonçalvès D., Cerezo M., Marchetti S. (2015). The PRKAA1/AMPKα1 pathway triggers autophagy during CSF1-induced human monocyte differentiation and is a potential target in CMML. Autophagy.

[61] Ma D., Lian F., Wang X. (2019). PLCG2 promotes hepatocyte proliferation in vitro via NF-κB and ERK pathway by targeting bcl2, myc and ccnd1. Artif. Cells Nanomed. Biotechnol..

[62] Baird L., Dinkova-Kostova A.T. (2011). The cytoprotective role of the Keap1-Nrf2 pathway. Arch. Toxicol..

[63] Sun Z., Huang Z., Zhang D.D. (2009). Phosphorylation of Nrf2 at multiple sites by MAP kinases has a limited contribution in modulating the Nrf2-dependent antioxidant response. PLoS ONE.

[64] Baird L., Yamamoto M. (2020). The Molecular Mechanisms Regulating the KEAP1-NRF2 Pathway. Mol. Cell Biol..

[65] Taguchi K., Motohashi H., Yamamoto M. (2011). Molecular mechanisms of the Keap1–Nrf2 pathway in stress response and cancer evolution. Genes. Cells.

[66] Bai Y., Guo N., Xu Z., Chen Y., Zhang W., Chen Q., Bi Z. (2023). S100A1 expression is increased in spinal cord injury and promotes inflammation, oxidative stress and apoptosis of PC12 cells induced by LPS via ERK signaling. Mol. Med. Rep..

[67] Chen Y., Jiang L., Li M., Shen Y., Liu S., Yang D. (2024). Huanglian Jiedu decoction alleviates neurobehavioral damage in mice with chronic alcohol exposure through the RAS-RAF-MEK-ERK pathway. Heliyon.

[68] Lai Q., Xie T., Huang Y. (2023). Inhibitory effect of miR-27b-3p and Nrf2 regulation on metabolic memory formation in human RPE cells. Chin. J. Exp. Ophthalmol..

[69] Lian B., Gu J., Zhang C., Zou Z., Yu M., Li F., Wu X., Zhao A.Z. (2022). Protective effects of isofraxidin against scopolamine-induced cognitive and memory impairments in mice involve modulation of the BDNF-CREB-ERK signaling pathway. Metab. Brain Dis..

[70] Zhang W., Geng X., Dong Q., Li X., Ye P., Lin M., Xu B., Jiang H. (2023). Crosstalk between autophagy and the Keap1-Nrf2-ARE pathway regulates realgar-induced neurotoxicity. J. Ethnopharmacol..

[71] Zhang L., Guo Y., Wang H., Zhao L., Ma Z., Li T., Liu J., Sun M., Jian Y., Yao L. (2019). Edaravone reduces Aβ-induced oxidative damage in SH-SY5Y cells by activating the Nrf2/ARE signaling pathway. Life Sci..

[72] Salakou S., Kardamakis D., Tsamandas A.C., Zolota V., Apostolakis E., Tzelepi V., Papathanasopoulos P., Bonikos D.S., Papapetropoulos T., Petsas T.G. (2007). Increased Bax/Bcl-2 ratio up-regulates caspase-3 and increases apoptosis in the thymus of patients with myasthenia gravis. In Vivo.

[73] Lu X. (2022). Structure and Function of Ligand CX3CL1 and its Receptor CX3CR1 in Cancer. Curr. Med. Chem..

[74] Subbarayan M.S., Joly-Amado A., Bickford P.C., Nash K.R. (2022). CX3CL1/CX3CR1 signaling targets for the treatment of neurodegenerative diseases. Pharmacol. Ther..

[75] Liu Z., Condello C., Schain A., Harb R., Grutzendler J. (2010). CX3CR1 in microglia regulates brain amyloid deposition through selective protofibrillar amyloid-β phagocytosis. J. Neurosci..

[76] Cook D.N., Chen S.C., Sullivan L.M., Manfra D.J., Wiekowski M.T., Prosser D.M., Vassileva G., Lira S.A. (2001). Generation and analysis of mice lacking the chemokine fractalkine. Mol. Cell Biol..

[77] Jian Z., Nonaka I., Hattori S., Nakamura S. (1996). Activation of ras and protection from apoptotic cell death by BDNF in PC12 cells expressing trkB. Cell. Signal..

[78] Koffie R.M., Hyman B.T., Spires-Jones T.L. (2011). Alzheimer’s disease: Synapses gone cold. Mol. Neurodegener..

